# 35 Years of GLP in Germany: A Brief Overview About Its Successful History

**DOI:** 10.1007/s43441-026-01009-8

**Published:** 2026-07-17

**Authors:** Sebastian Schmidtsdorff, Christian Strüh

**Affiliations:** GLP Federal Bureau, German Federal Institute for Risk Assessment, Max- Dohrn-Str. 8-10, 10589 Berlin, Germany

**Keywords:** Good laboratory practice (GLP), GLP Federal Bureau, Germany, Compliance monitoring, OECD, Non-clinical safety studies, Mutual acceptance of data (MAD), Inspection

## Abstract

Good Laboratory Practice (GLP) emerged in the 1970s in response to serious deficiencies in the validity and reproducibility of preclinical safety data submitted in the USA. Non-adherence to scientific standards in the use of laboratory methods and the data generated, and even fraudulent practices, led to the establishment of binding regulatory standards, which were later adopted by the OECD as the “Principles of GLP”, alongside the Council Decision on the Mutual Acceptance of Data (MAD). This framework, expanded to include compliance monitoring programmes and non-member country participation and has since formed the foundation for international harmonization of non-clinical safety testing. Germany, as an early adopter of the OECD GLP system, provides a prominent example of long-term implementation. Since the national compliance monitoring programme became legally binding in 1990, thousands of inspections were carried out in the meantime. German test facilities cover all OECD-defined areas of expertise and the German compliance monitoring authorities maintain rigorous inspection cycles in line with OECD recommendations, with a notable share operating for decades within the programme. This long-standing infrastructure in Germany contributes to the reliable and robust application of the OECD’s GLP principles. This article presents a comprehensive overview of the historical development, international transposition and national application of GLP, using Germany as a case study after 35 years of implementation. By compiling regulatory milestones, structural data, and long-term monitoring outcomes, the publication aims to provide a transparent evidence base for assessing the effectiveness of GLP frameworks. The systematic presentation of these data serves both to document Germany’s contribution to the international GLP landscape and to support ongoing discussions on harmonization, resource allocation, and future challenges in non-clinical safety assessment.

## Introduction

### History of GLP

In the 1970s, concerns about the validity and reproducibility of preclinical safety data submitted for medicinal products to the U.S. Food and Drug Administration (FDA) prompted the formalization of binding minimum industry standards. Subsequent inspections revealed widespread issues, including poor laboratory practices like inadequate study planning and personnel training, deficient laboratory equipment, execution errors, lack of documentation, resulting in questionable study validity and robustness. Even fraudulent activities were observed, such as the undocumented replacement of dead animals and manipulation of data to match final reports [1–3]. Particularly noteworthy cases of systematic misconduct are the findings revealed during the inspections at Industrial Bio-Test Laboratories (IBT) and G.D. Searle & Company (Searle) that were made public during the Kennedy Hearings in the U.S. Congress [4, 5]. These revelations led to the FDA’s publication of proposed Good Laboratory Practice (GLP) regulations in 1976, with the final rules becoming effective in June 1979 (21 CFR 58). These regulations aimed to ensure the accuracy and integrity of study reports submitted in the USA together with an implementation of a pilot inspection programme to check for compliance with the proposed regulations [1, 2]. Concurrently, the U.S. Environmental Protection Agency (EPA) faced similar issues in chemical and pesticide studies, prompting it to issue its own GLP regulations in the early 1980s [6, 7].

The issues identified in the USA spurred international efforts to standardize GLP practices. In 1978, the Organisation for Economic Co-operation and Development (OECD) established an Expert Group under the Special Programme on the Control of Chemicals to develop the OECD Principles of GLP, which were based on the FDA’s 1976 regulations. These principles were formally recommended for use in member countries by the OECD Council in 1981 through the “Decision Concerning the Mutual Acceptance of Data in the Assessment of Chemicals” [C(81)30(Final)] [8]. This decision officially stipulated that data generated in compliance with OECD test guidelines and GLP principles in one member country should be accepted by other member countries for the assessment of chemicals, facilitating international trade and eliminating the need for duplicative testing [3].

To ensure compliance with GLP, the OECD emphasized the importance of national compliance monitoring programmes, which include laboratory inspections and study audits. The 1989 OECD Council “Decision-Recommendation on Compliance with Principles of Good Laboratory Practice” [C(89)87/FINAL] [9] required member countries to establish procedures for monitoring GLP compliance and to exchange information on the compliance status of their local test facilities. Over time, the OECD principles of GLP have been incorporated into the national legislation of several member countries and regions [7, 10–12], including the European Union (EU), which formally introduced the principles [13, 14] into European law through Commission Directive 87/18/EEC from 1986 (now directive 2004/10/EC) [15] and the inspection and verification of GLP through Council Directive 88/320/EEC from 1988 (now directive 2004/9/EC) [16].

The mutual acceptance of GLP-compliant data is a critical aspect of international regulatory frameworks. It ensures that laboratory studies are conducted in accordance with GLP as well as the OECD test guidelines and that comparable data quality is accepted across borders, thus preventing the unnecessary repetition of studies, reducing unnecessary animal testing and saving costs, and facilitating global trade [12]. The OECD has also worked to extend these principles beyond its member countries. In 1997, the OECD Council adopted the “Council Decision concerning the Adherence of Non-member Countries to the Council Acts related to the Mutual Acceptance of Data in the Assessment of Chemicals” (C(97)114/FINAL) [17], allowing non-member countries to voluntarily adhere to OECD GLP standards and to join the Mutual Acceptance of Data (MAD) system. These countries are called “provisional adherents” or “full adherents” [18]. Full adherents are non-member countries with implemented OECD GLP principles in their national legislation, evaluated by the OECD, with a recognized national GLP compliance monitoring programme (CMP), thus fully participating in the MAD system. Provisional adherents on the other hand are non-member countries in the process of becoming a full adherent, without a recognized national GLP CMP. Therefore, member countries and full adherents are not obliged to accept data from provisional adherents until they become full adherents. The inclusion of these adherents fosters broader international cooperation and acceptance of GLP-compliant data, further reducing trade barriers and promoting global public health and environmental safety [2, 19].

Despite these advances, challenges remain in the global implementation of GLP. In some developing countries, where costs for GLP studies are lower [11] and laboratory capacities sometimes higher, national compliance monitoring authorities (CMAs) may not be fully established, properly empowered and therefore not officially recognized, yet these countries often have significant chemical and pharmaceutical industries that produce preclinical safety data [2, 7, 11]. In such cases, GLP inspectors from member countries with established GLP monitoring systems may inspect individual non-clinical safety studies and test facilities to ensure compliance with international standards in those so-called “non-adherents”. However, these studies are not subject to the MAD; rather, the inspections are conducted in the national interest to support the inspecting countries’ regulatory procedures.

In conclusion, the establishment and global adoption of GLP have been pivotal in ensuring the reliability and integrity of non-clinical safety studies, protecting public health, and facilitating international trade. The ongoing efforts to harmonize GLP standards and compliance monitoring across both OECD member and non-member countries underscore the importance of GLP in the global regulatory landscape.

### What is GLP?

Good Laboratory Practice is a quality system concerned with the organizational process and the conditions under which non-clinical health and environmental safety studies are planned, performed, monitored, recorded, archived and reported. This is the definition of GLP in the “OECD Principles of Good Laboratory Practice” (written down in the so-called “OECD GLP Document No. 1”) [13], which was transposed into German law as Annex 1 of the German ChemG [20]. Test items to be evaluated under GLP can vary from pharmaceuticals, veterinary drugs, pesticides and biocides, cosmetic products, food and feed additives as well as industrial chemicals. Depending on the national jurisdiction, GLP may also be applied to detergents, medical devices or other regulated products [13].

The OECD GLP principles clearly define the responsibilities of key personnel within a test facility, including test facility management, the study director, the study personnel and the quality assurance. Each of these individuals plays a critical role in ensuring the reliability, integrity and reconstruct ability of the study data over the entire raw data lifecycle. Furthermore, requirements regarding the adequacy and maintenance of facilities and equipment to ensure the proper conduct of studies are laid down. These elements collectively ensure the scientific and GLP-compliant credibility of non-clinical safety studies submitted for regulatory purposes.

As a consequence, the widespread international implementation of the GLP principles and the largely mutual recognition of study data have contributed more than any other quality system to health, environmental and animal protection. Multinational companies can, for instance, cut back their animal experiments and costs by around 70% thanks to the mutual recognition of GLP implementation [21].

### Overview of the German GLP Compliance Monitoring System

In the Federal Republic of Germany (at that time often referred to as “West Germany”), the Federal Government recommended the voluntary application of the OECD GLP principles [13] at an early stage in March 1983, long before they became legally binding, in order to ensure the recognition of German approvals on the U.S. market. Until 1989, however, inspections for non-clinical safety studies were almost exclusively applied for drugs and medicinal products by the industry [22].

Since the German Democratic Republic (GDR, commonly referred to as “East Germany”) was not a member of the OECD and had no bilateral agreements with the U.S. regarding the recognition of non-clinical safety data, GLP principles were not established by law in the GDR until the German reunification.

With the revision of the German Chemicals Act (ChemG) in August 1990 [20], the EU directives became legally effective and were incorporated into the pre-existing GLP system. The German GLP monitoring system then started its mandatory activity on the basis of the newly introduced “General Administrative Provision on the Procedure of National Monitoring to ensure Good Laboratory Practice” (ChemVwV-GLP) in October 1990 [23]. This ChemVwV-GLP specifies the OECD guidelines [14, 24] and recommendations for monitoring compliance by the competent CMAs and transposes them into German law.

Monitoring in the Federal Republic of Germany, which is a full member and co-founder of the OECD, is carried out by the CMAs of the 16 federal states of Germany, which inspect and certify local facilities to ensure that they comply with GLP requirements. In addition, the GLP Federal Bureau at the German Federal Institute for Risk Assessment is the competent CMA for federal GLP facilities, who conduct safety studies on behalf of federal ministries, and is also inspecting laboratories in non-adherent, non-member countries outside the OECD’s MAD system [25]. The German GLP Federal Bureau is also responsible for coordination and harmonization of GLP-relevant tasks in Germany and for dissemination of information at the national and international level, stipulated in §§ 19c and 19d ChemG [20]. Pursuant to the ChemG, it annually reports on adherence to the GLP principles in German test facilities (a location where non-clinical health and environmental safety studies are carried out) and test sites (a location where only phases of such a study are conducted) to the EU and OECD on the basis of the inspections carried out.

Together, the 17 German CMAs form one united, harmonized and holistic national CMP, which was last successfully evaluated by the OECD in 2018. During German GLP inspections, no distinction is made as to whether the laboratories and the test items they examine (pharmaceuticals, veterinary drugs, pesticides and biocides, cosmetic products, food and feed additives as well as industrial chemicals) are assigned to a specific area. According to § 19b para 1 of the Chemicals Act (ChemG), German CMAs will only inspect test facilities or test sites upon request and if GLP studies (or phases of studies) are carried out.

**Fig. 1:**
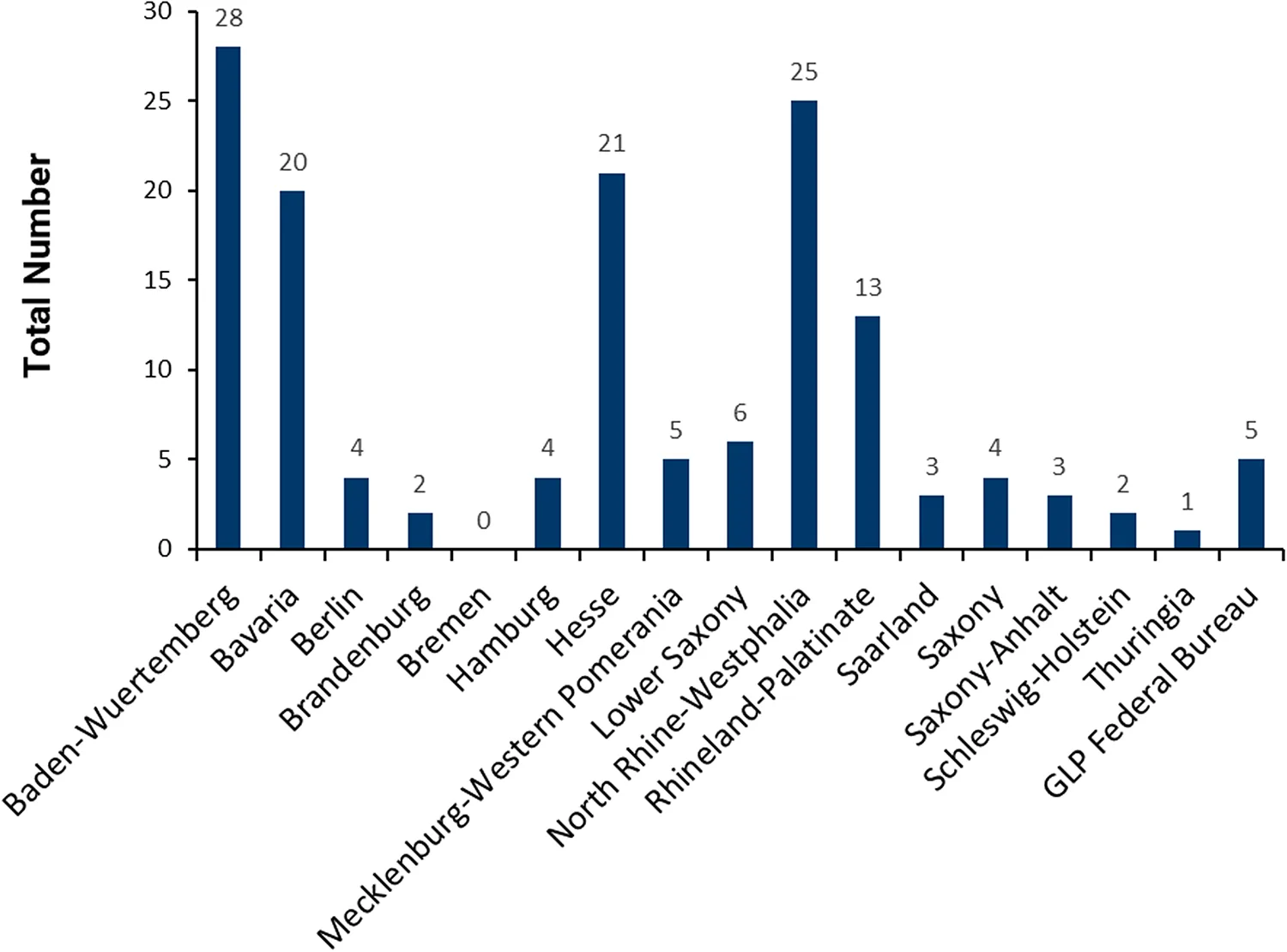
Current domestic and non-MAD test facilities and test sites (*N* = 146) within the German GLP Compliance Monitoring Programme of each federal state CMA and the GLP Federal Bureau (as of 31 December 2024)

**Fig. 2:**
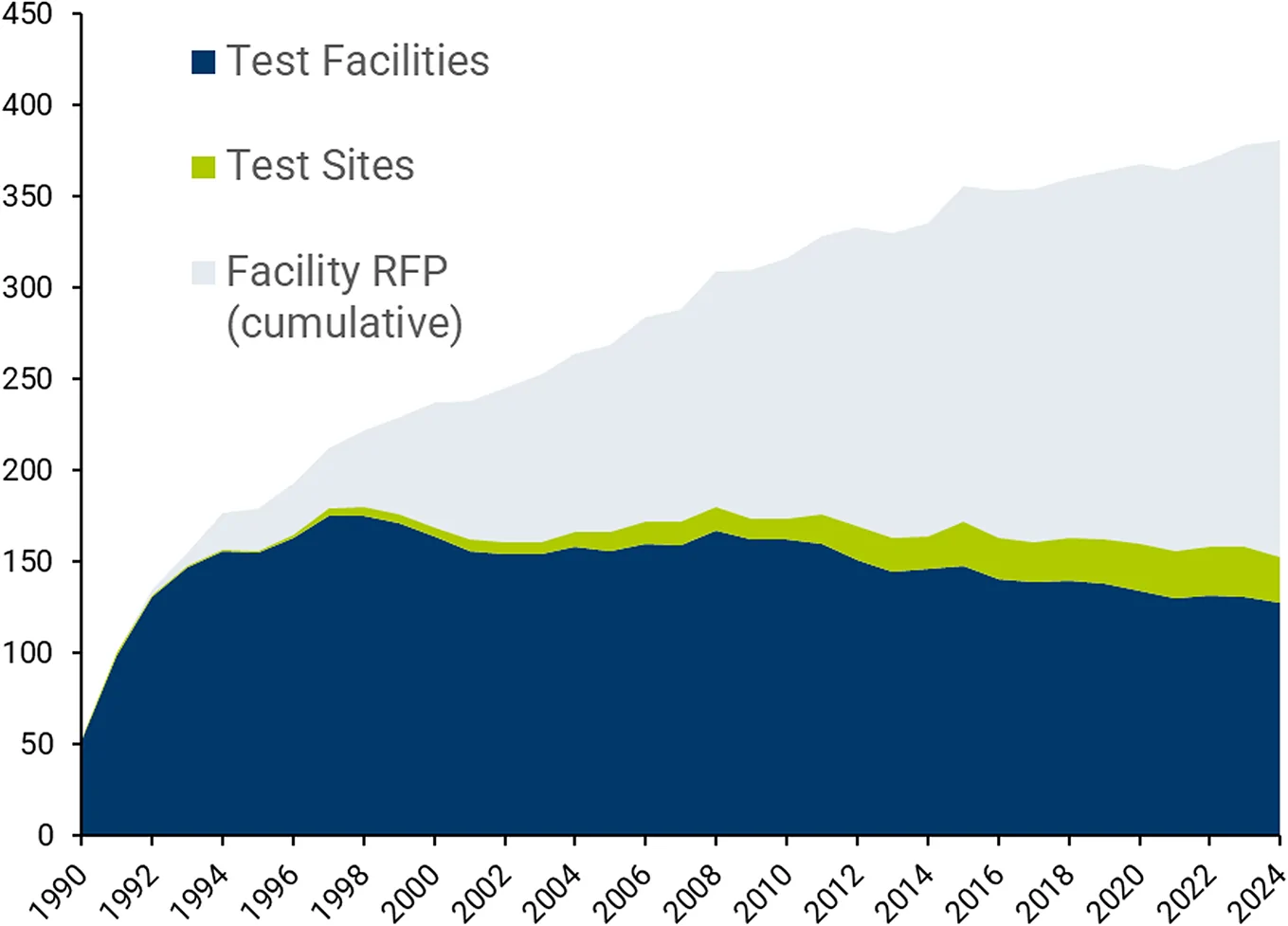
Number of test facilities and test sites in the German compliance monitoring programme per year (stacked)

As of 31 December 2024, a total number of 143 test facilities and test sites are operating within the German CMP plus three test facilities in the P. R. China under the monitoring of the German GLP Federal Bureau (Fig. 1). This number was relatively constant over the last 35 years (Fig. 2), even though facilities were removed from programme (RFP), since new GLP laboratories started operating in Germany. A minor decrease of test facilities can be observed, however, the impression that fewer GLP studies are being carried out at the same time is deceptive, as the total number of highly specialized test sites is constantly increasing.

**Fig. 3:**
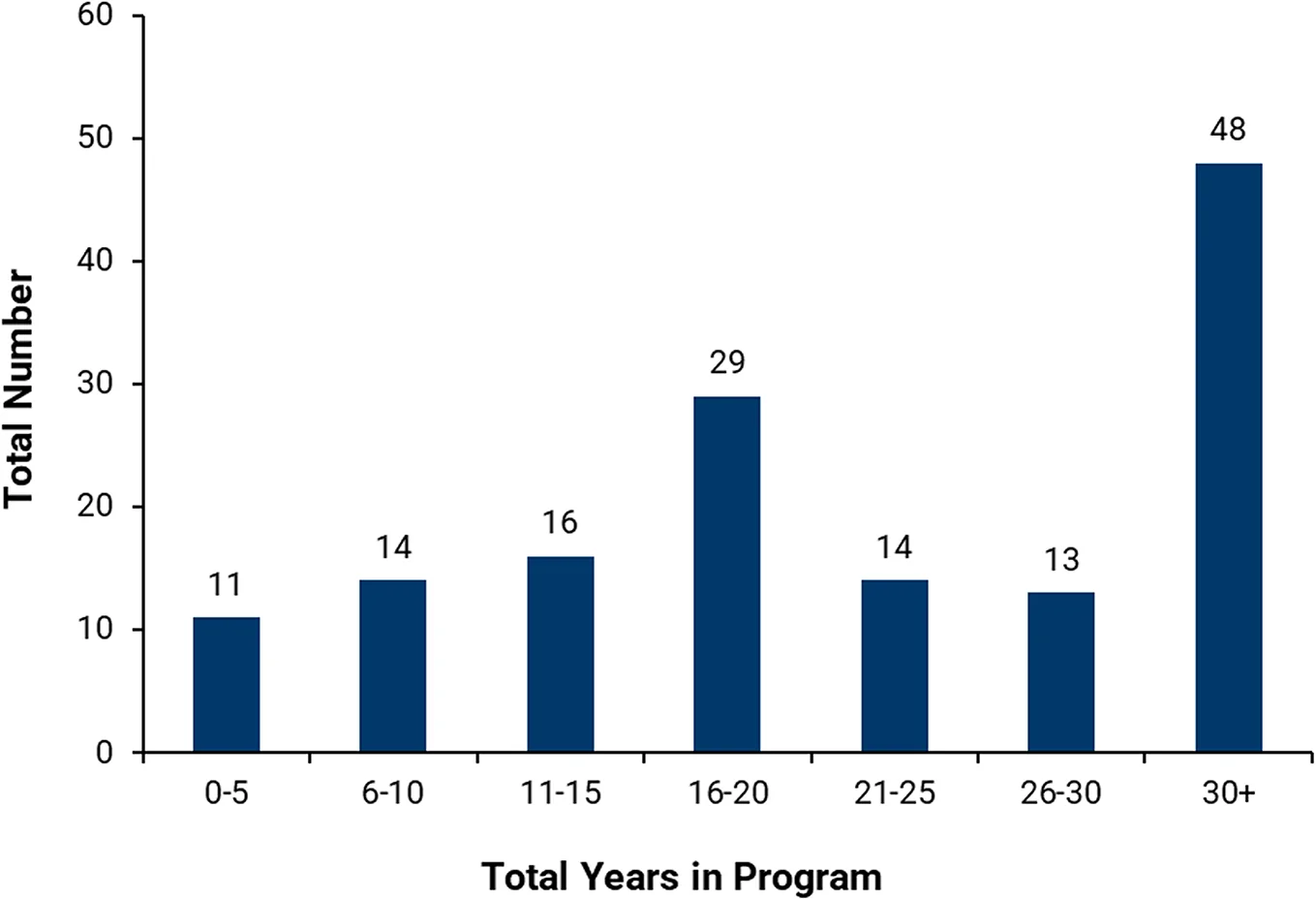
Duration of test facilities and test sites currently within German Compliance Monitoring Programme

**Fig. 4:**
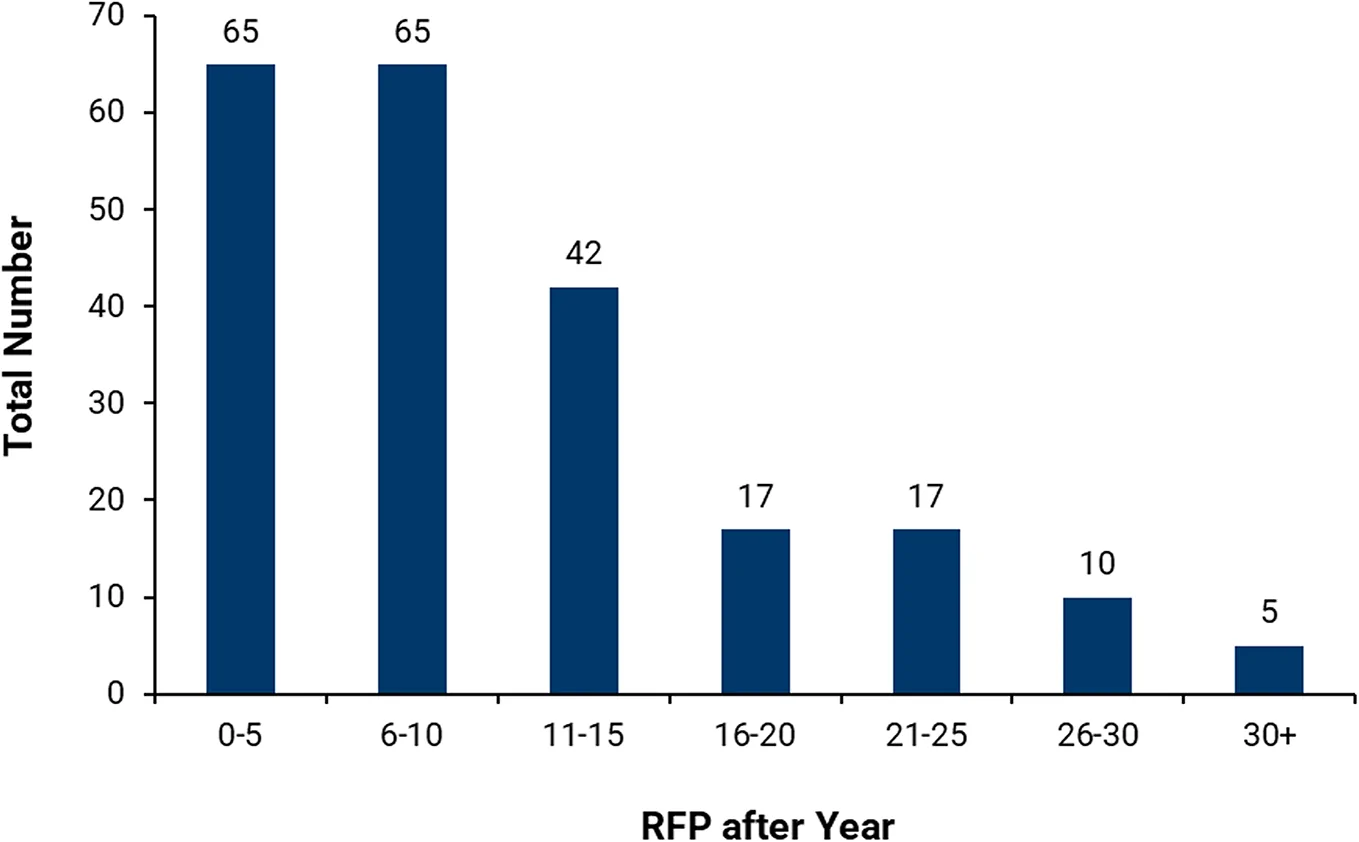
Duration of GLP operation for German test facilities and test sites Removed from Programme (RFP)

Around one third of all German facilities currently within the CMP have been part of the German GLP system since its inception and around two thirds are now operating for more than 15 years (Fig. 3). If facilities leave the GLP monitoring programme permanently, this RFP occurs on average after 11 years (statistical mean, see also Fig. 4). It can therefore be stated that Germany has a very high number of test facilities that have been in the business for a long time and play a decisive role in GLP studies in the international registration process.

**Fig. 5:**
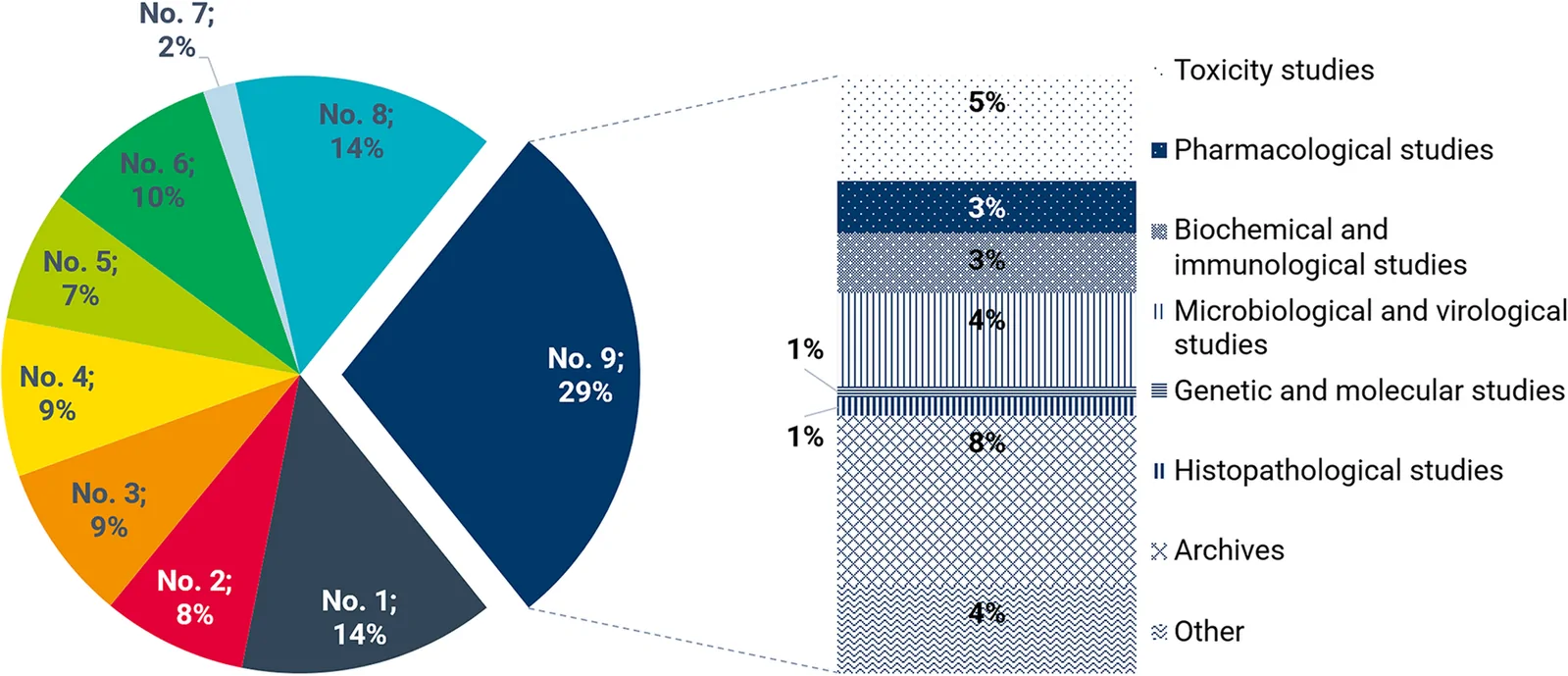
Area of Expertise (AoE) for German test facilities and test sites currently within GLP Compliance Monitoring Programme (note: AoE no. 9 is used for studies that are not categorized directly under AoE 1–8 or to limit an AoE, such as toxicity studies, to specific test methods within that AoE). A detailed description of AoE 1–9 is provided below Fig. 7

**Fig. 6:**
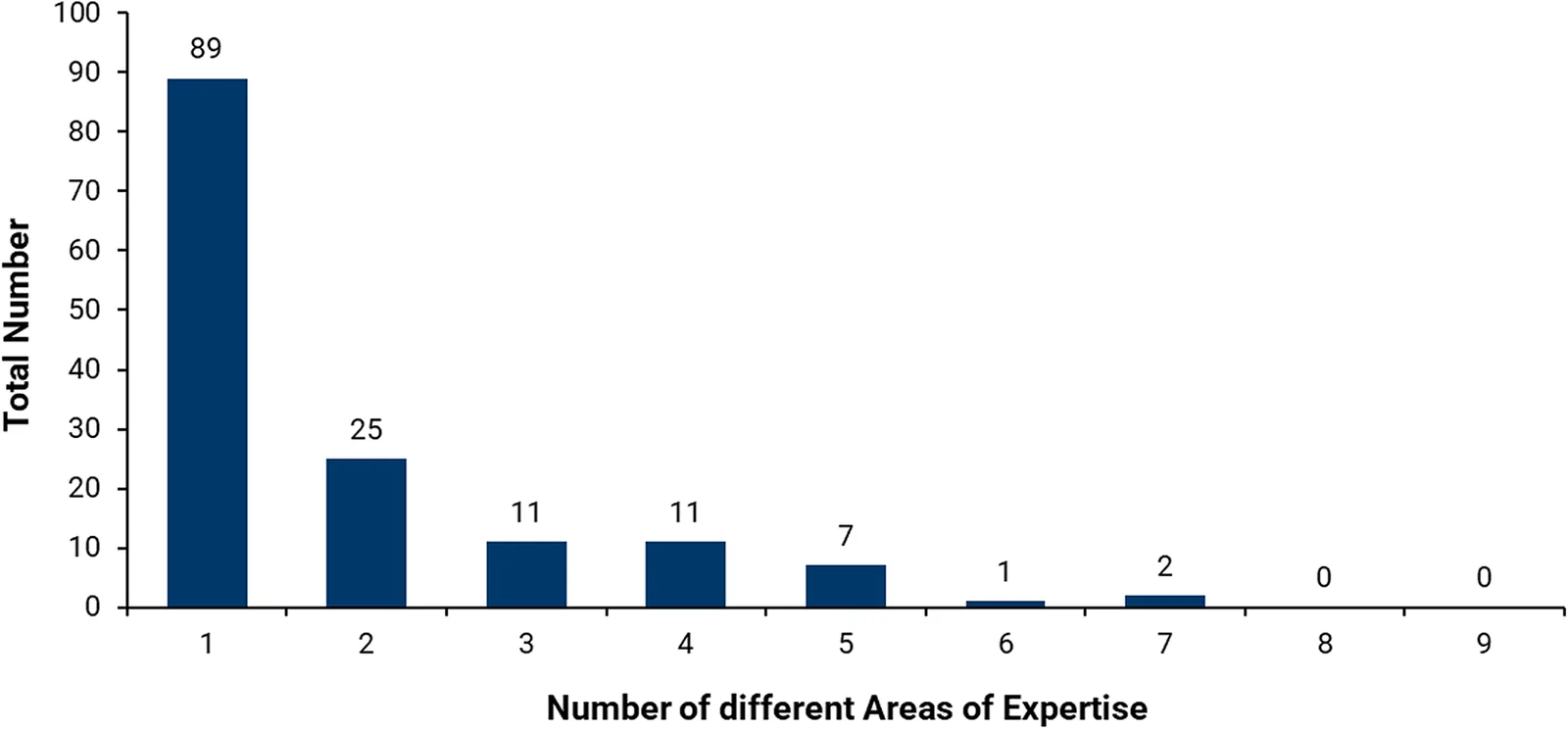
Number of different AoE per test facility or test site

All areas of expertise, and thus the full range of preclinical safety studies as listed in OECD GLP Document No. 2 (“Revised Guides for Compliance Monitoring Procedures for Good Laboratory Practice”) [14], are covered comprehensively and evenly by all test facilities in Germany, except for area of expertise No. 7 (“studies on effects on mesocosms and natural ecosystems”), which is only conducted by a few facilities (Fig. 5). Facilities are typically (78%; *N* = 114) certified for one or two GLP AoEs (Fig. 6) [25].

**Fig. 7:**
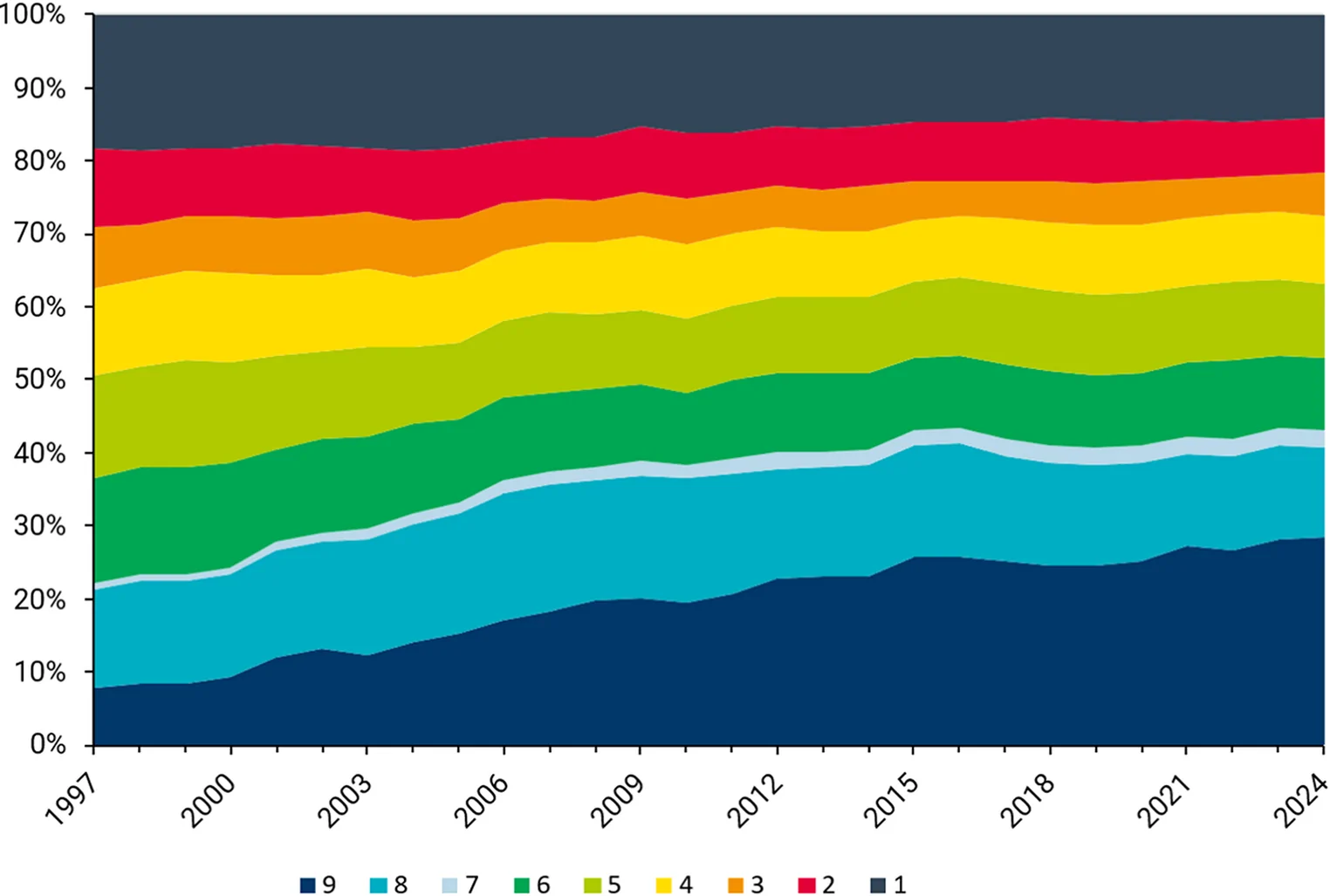
Distribution of different AoEs since 1997 in Germany (1 = physical-chemical testing; 2 = toxicity studies; 3 = mutagenicity studies; 4 = environmental toxicity studies on aquatic and terrestrial organisms; 5 = studies on behaviour in water, soil and air, bioaccumulation; 6 = residue studies; 7 = studies on effects on mesocosms and natural ecosystems; 8 = analytical and clinical chemistry testing; 9 = other studies)

Over time, it is apparent that the number of laboratories with area of expertise No. 9 (“other”, has to be specified by the inspecting CMA) has increased significantly. This category is usually used for highly specialized laboratories with a focus on certain sub-areas that are not directly covered by the other eight OECD AoEs or if such an AoE is to be limited to certain tests. Apart from this, the distribution of all other areas has remained roughly the same (Fig. 7).

Archiving of GLP studies, as it represents a relevant (final) lifecycle step of a study, and facility-related GLP documents are activities that must be carried out under GLP. According to Annex 1, Sect. 2, No. 10 of the ChemG, the archive period is 15 years in Germany. A distinctive feature of the German GLP system is the possibility to apply for this under area of expertise No. 9. Therefore, specialized archive service providers can also be GLP certified, if they adhere to the relevant GLP principles for archiving, but are then only considered a test site and not a test facility, as no studies are conducted there.

**Fig. 8:**
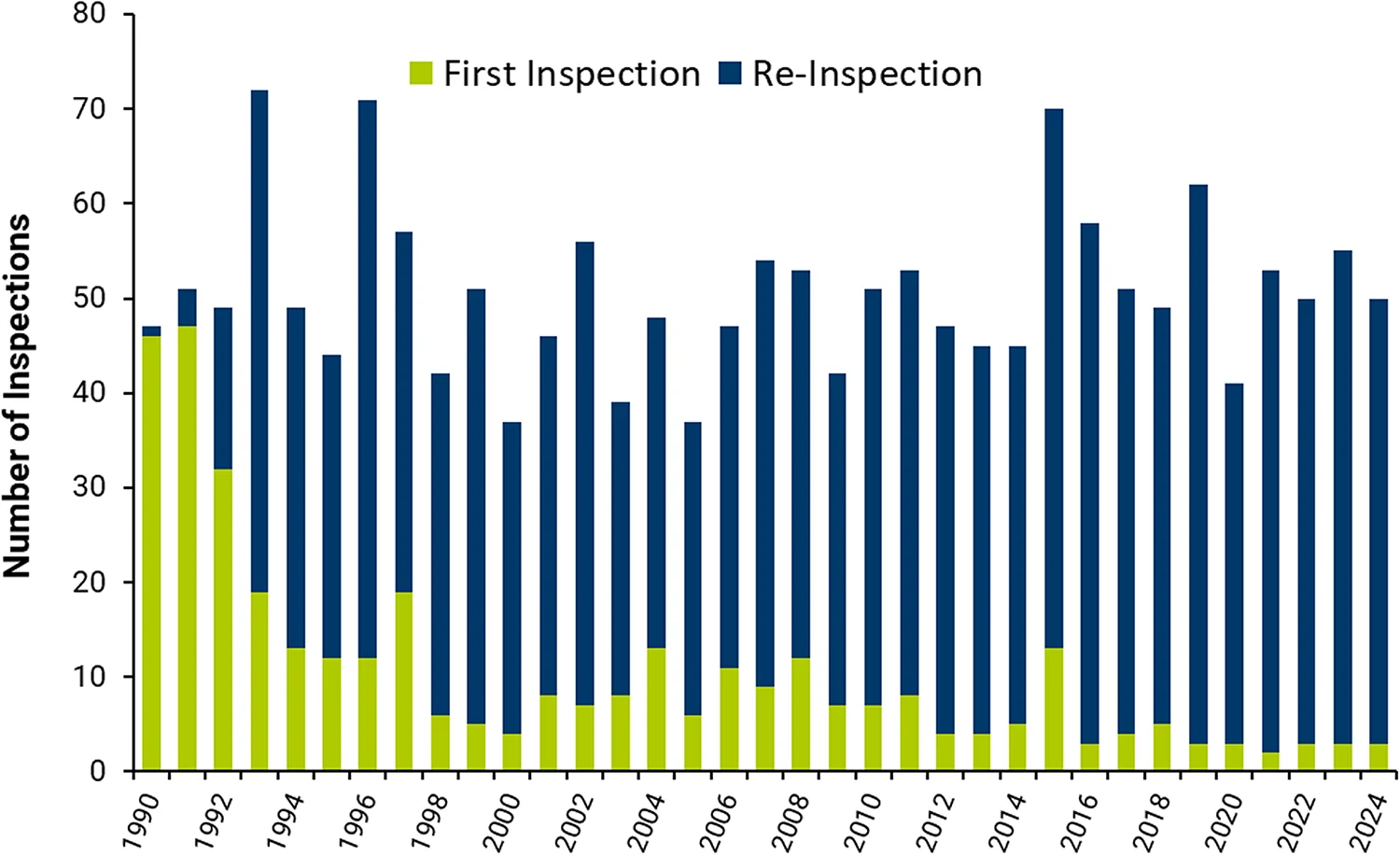
Number of GLP inspections per year in Germany

**Fig. 9:**
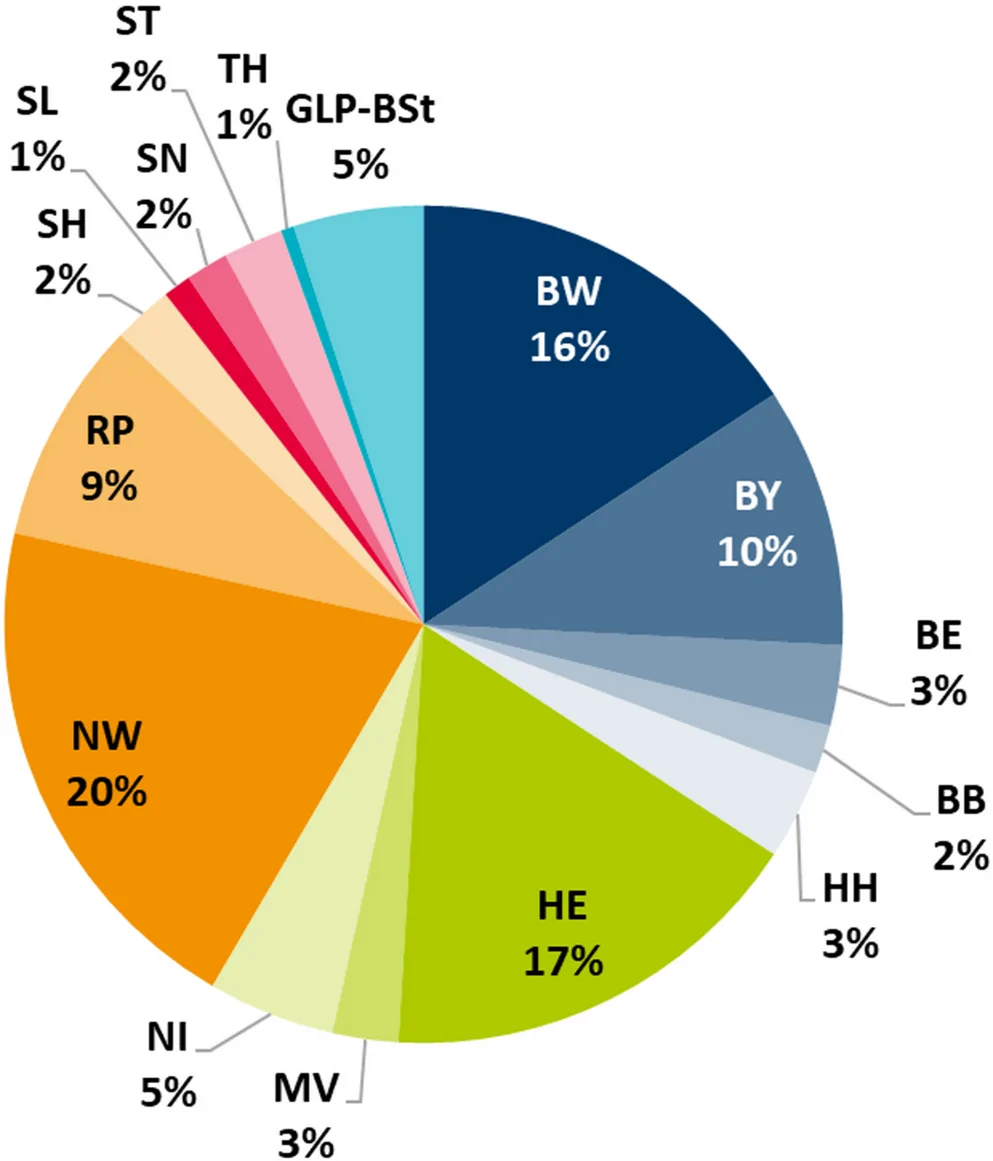
Percentage of GLP inspection since 1990 performed by each CMA in Germany (BW = Baden-Württemberg, BY = Bavaria, BE = Berlin, BB = Brandenburg, HH = Hamburg, HE = Hesse, MV = Mecklenburg-Western Pomerania, NI = Lower Saxony, NW = North Rhine-Westphalia. RP = Rhineland-Palatinate, SH = Schleswig-Holstein, SL = Saarland, SN = Saxony, ST = Saxony-Anhalt, TH = Thuringia, GLP-BSt = GLP Federal Bureau)

Since implementation of the national CMP in Germany, a total of 1968 inspections were performed, not including specific study audits requested by receiving authorities such as the EMA, ECHA or EFSA. In the last years, approximately 50–60 inspections per year are conducted (Fig. 8) by all 17 CMAs, of which are approximately three first inspections per year, even during the corona pandemic years. As the majority of these facilities are located in the western and southern part of Germany, the five federal states Baden-Wuertemberg (BW), Bavaria (BY), Hesse (HE), North Rhine-Westphalia (NW) and Rhineland-Palatinate (RP) are correspondingly responsible for approximately 72% of the annual inspections (Fig. 9).

**Fig. 10:**
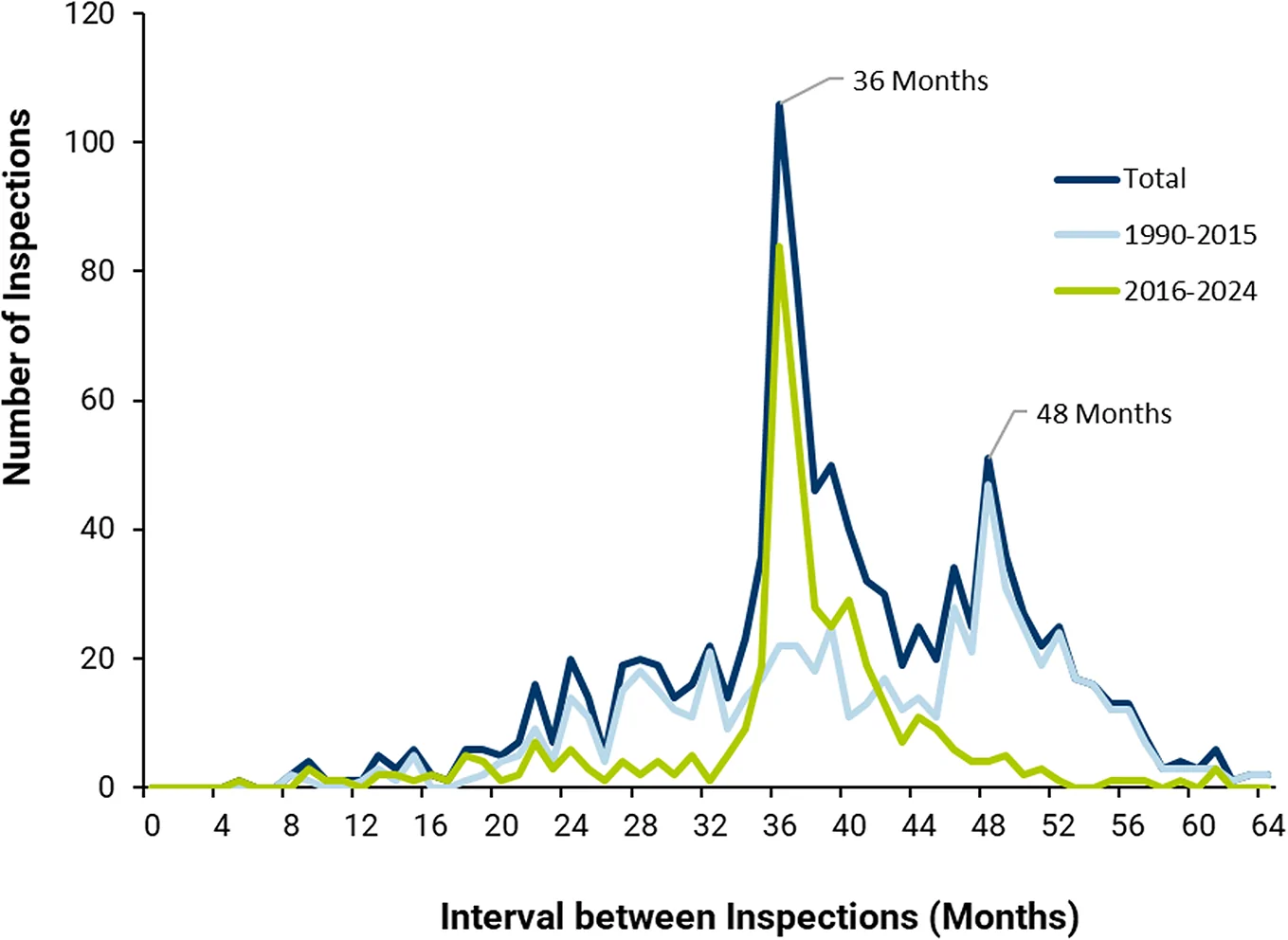
Histogram of German re-inspection intervals since 1990 (that the graph from 2016 onwards was chosen to show only inspections that no longer fall under the administrative regulation prior to 2011, as the old inspection cycle could still be applied once after 2011, provided that the re-inspection was requested by 2011)

Due to the fact that German test facilities and test sites operate for a long time before they are RFP, each company is statistically inspected 5 times with a mean re-inspection interval of 3 years. As displayed by Fig. 10, two peaks can be observed in the histographical distribution chart of the re-inspection intervals. The highest abundance for re-inspections is shown at an interval of 36 months, a second peak at 48 months. This second peak is due to the fact that the mandatory interval for an application for re-inspection in the German ChemVwV-GLP was shortened from 4 years to 3 years in 2011. This took into account a recommendation, made following an evaluation by representatives of the OECD GLP Working Party, to reduce the average inspection interval to less than 3½ years [26] and has resulted in significantly shorter and more reproducible inspection intervals.

## Conclusions

Over the past 50 years, the establishment and operation of GLP have fundamentally transformed the conduct of non‑clinical safety studies. Germany, a member of the OECD GLP system from its beginning, has developed and contributed extensive compliance monitoring experience to increase the robustness of the GLP framework. Following the voluntary adoption of the OECD GLP principles in 1983 and their establishment by law in 1990, it now comprises a coherent and comprehensive compliance monitoring network of 143 national GLP testing facilities and three affiliated international test facilities. This sustained commitment, which is manifested in the activities and key figures presented, has consolidated Germany’s position in the OECD’s GLP system. At the same time, the country-specific implementation of GLP regulations, the conduct of compliance monitoring in other countries and the spread of new testing areas, New Approach Methodologies (NAMs) and techniques underline the need for on-going harmonization and strategic allocation of resources.

**Fig. 11:**
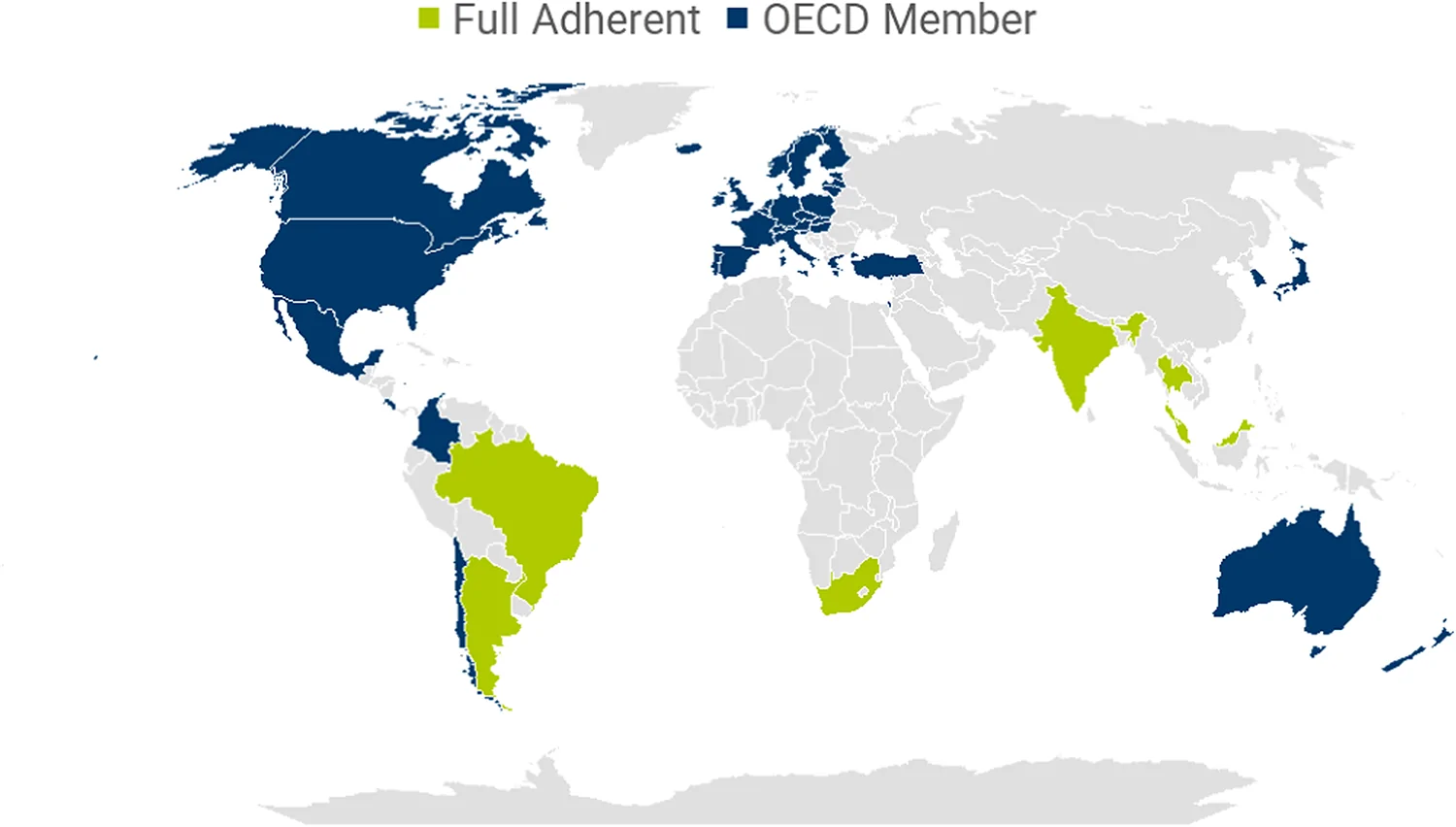
National GLP Compliance Monitoring Programmes participating in the Mutual Acceptance Data (MAD) system (as of 2025)

Looking ahead, GLP remains central to ensuring the reliability, integrity, and transparency of non‑clinical safety studies worldwide. Continued OECD oversight and targeted support for emerging GLP CMAs will be critical to sustaining MAD and to address novel scientific and regulatory challenges. Currently, in 2025, 45 countries, representing 40% of the world’s population, participate in the MAD system, either as OECD members or as full adherents (Fig. 11) [18]. This number must be increased further in the future to meet the ambition of a global system.

Ultimately, the OECD GLP framework’s success will depend on both unwavering commitment from regulators and inspectors, as well as the willingness of scientific test facilities to embrace its principles as essential safeguards for data integrity and the reliability of submitted studies. This combination will serve as cornerstones for science and compliance.

## Methods

According to the German Chemicals Act, the GLP Federal Bureau is receiving reports (§ 19b para 3 and § 19c para 1) of every inspection and study audit conducted by the 16 federal CMAs. An updated list of all test facilities and test sites currently in the German GLP CMP is then published for the general public [25]. Based on the reports, which has been collected for over 35 years, an anonymized list of test facilities and test sites, their area(s) of expertise, their local CMA and inspection dates was generated. This list contains approx. 3000 entries that were filtered for inspection and RFP events. All calculations and depictions are only based on these filtered data.

As the GLP Federal Bureau does not have a mandate to publish this raw data, even in anonymized form, it cannot be made available as supplementary material.

## Data Availability

As the German GLP Federal Bureau does not have a mandate to publish the raw data, even in anonymized form, they cannot be made available as supplementary material. Nevertheless, the accuracy and quality of the underlying data can be guaranteed as fully trustworthy, as it is reviewed annually by all relevant local compliance monitoring authorities and must also be submitted to the EU and the OECD.
